# CLIP-EZ: a computational tool for HITS-CLIP data analysis

**DOI:** 10.1186/1471-2105-15-S10-P1

**Published:** 2014-09-29

**Authors:** Xue Zhong, Qi Liu, Yu Shyr

**Affiliations:** 1 Center for Quantitative Sciences, Vanderbilt University School of Medicine, Nashville, TN 37232 USA

## Background

Mapping the binding regions of mRNA-binding proteins is critical to the understanding of their regulatory roles in cellular processes. Recent development in experimental technologies combines high throughput sequencing with crosslink immunoprecipitation (HITS-CLIP), which has the merit of detecting RNA-protein interaction sites at a high resolution to single nucleotide level. Analysis of such data typically involves many steps, and teasing out true signals from noise requires crosslink induced mutations (CIMS) analysis, peak identification, integration of the two signal types, and some downstream analysis such as motif finding and conservation evaluation. To our knowledge, there is a lack of a single computational tool that can perform all the tasks as mentioned in an easily accessible manner. Despite the fact that there are several tools available, each performs an individual task.

## Materials and methods

To facilitate the analysis task, we developed a command line tool that implements the analysis pipeline and outputs all relevant results, intermediate and final, with command line parameters specified by the user.

## Results

The outputs are comprehensive which include the map of binding regions, pie charts for the annotation of binding sites, a list of high confidence binding regions which are annotated for CIMS footprint, peak height, motif, and conservation score and ranked by a score that integrates information across the annotations. The output also provides a bed file that can be loaded into the UCSC genome browser for visualization. This software can also be used to identify differential binding regions across biological conditions. This command line tool will be made available for download from GitHub.

